# Global trends in clinical trials involving pluripotent stem cells: a systematic multi-database analysis

**DOI:** 10.1038/s41536-020-00100-4

**Published:** 2020-09-11

**Authors:** Julia Deinsberger, David Reisinger, Benedikt Weber

**Affiliations:** 1 Disease Modeling and Organoid Technology (DMOT) Research Group, Department of Dermatology, Medical University of Vienna, Vienna, Austria; 2 Skin and Endothelium Research Division (SERD), Department of Dermatology, Medical University of Vienna, Vienna, Austria

**Keywords:** Embryonic stem cells, Induced pluripotent stem cells

## Abstract

Pluripotent stem cells (PSCs) hold great potential for novel therapeutic approaches to regenerate or replace functionally impaired tissues. Since the introduction of the induced pluripotent stem cell technology in 2006, the number of scientific publications on this topic has constantly been increasing. However, so far no therapy based on PSCs has found its way into routine clinical use. In this study, we examined research trends related to clinical trials involving PSCs based on data obtained from ClinicalTrials.gov, the ICTRP database from the World Health Organization, as well as from a search of all individual databases that are included in the ICTRP using a multistep search algorithm. Following a stringent inclusion/exclusion procedure 131 studies remained that could be classified as clinical trials involving PSCs. The magnitude of these studies (77.1%) was observational, which implies that no cells were transplanted into patients, and only a minority of studies (22.9%) were of an interventional study type. The number of clinical trials involving induced pluripotent stem cells (iPSCs, 74.8%) was substantially higher than the one involving embryonic stem cells (ESCs, 25.2%). However, the picture changes completely when focusing on interventional studies, where in the majority (73.3%) of cases ESCs were used. Interestingly, also the study duration was significantly shorter for interventional versus observational trials (*p* = 0.002). When focusing on the geographical study regions, it became obvious that the greatest part of all observational trials was performed in the USA (41.6%) and in France (16.8%), while the magnitude of interventional studies was performed in Asian countries (China 36.7%, Japan 13.3%, South Korea 10.0%) and in the field of ophthalmology. In summary, these results indicate that only a limited number of trials were focusing on the actual transplantation of PSCs into patients in a rather narrow field of diagnoses. The future will tell us, if the iPSC technology will ultimately overcome the current challenges and will finally make its way into routine clinical use.

## Introduction

Pluripotent stem cells are defined by their self-renewal capacity and ability to differentiate into any cellular phenotype of the human body^1^. This plasticity has generated significant hope for therapies that may enable repair of functionally impaired tissues^2,3^. Several studies involving embryonic stem cells (ESCs) have been performed showing initial clinical success^4–6^. However, the use of ESCs is limited by several hurdles. Most importantly, the derivation of ESCs is associated with the destruction of the human embryo at the blastocyst-stage, which is associated with ethical concerns^7^. Secondly, ESCs are isolated from pre-implantation blastocysts and are thus, by their nature, never of autologous origin, which may cause immunological rejection by the host. The generation of induced pluripotent stem cells (iPSCs) alleviated these two major obstacles and thus holds the potential to open up completely new therapeutic options. The essence of iPSCs is that a mature, finally differentiated cell is reprogrammed via insertion of a set of genes, generating a pluripotent cell line for a specific patient^8^. Since the first report on iPSCs by Yamanaka et al. in 2006^9^, we have experienced publication of a plethora of studies involving preclinical in vitro^10,11^ and in vivo^12–16^ as well as first clinical studies in humans investigating the feasibility and safety of ESCs and iPSCs for therapeutic applications. Ongoing efforts to realize stem cell-based therapies for ophthalmic diseases and neurological disorders have so far shown promising results^5,12,17–19^. This rapid translation of the technology also supports the enormous potential and application range of pluripotent stem cell research^8,20,21^. However, the published information on trials is generally limited by a significant publication bias, implying that negative results may not be published at all. In addition, results may not be published due to a lack of interest by sponsoring institutions or they will be published in different languages. This is of particular interest as a growing body evidence has revealed genetic instability, epigenetic abnormalities and immunogenicity of iPSCs, raising safety concerns regarding clinical applications^22–28^. Additionally, pluripotent stem cells pose a considerable cancer risk in the recipient, firstly through potential teratoma formation and secondly due to genetic alterations as a consequence to the usage of integrating vectors creating a risk for reactivation of commonly used reprogramming factors (OCT4, SOX2, MYC, and KLF4), which are also highly expressed in various cancer types^25,29^.

The analysis of the ‘status quo’ of clinical trials on pluripotent stem cells would offer a comprehensive overview on the actual number and content of clinical trials involving this promising technology. Several studies focusing on the trends in stem cell research have been conducted so far^30–33^. However, these studies were either limited regarding the type of stem cell used, such as multipotent stem cells only^33^, or their search was conducted using only one database (mostly “ClinicalTrials.gov”)^30–33^. Even though “ClinicalTrials.gov”^34^ is the largest database for clinical trials and represented in over 200 countries around the globe, a search query in this database does not guarantee completeness. Therefore, we complemented our systematic search using the international clinical trials registry platform (ICTRP)^35^ from the Word Health Organization (WHO) and further individual databases contained in the ICTRP in order to achieve the most complete overview on clinical trials involving pluripotent stem cells. As part of this systematic analysis a particular focus was also put on the global distribution of the trials, the study type - observational versus interventional -, as well as on the number of patients enrolled.

## Results

### Results of the systematic multi-database search algorithm

In the database search using “ClinicalTrials.gov” 177 studies were identified when using the predefined search criteria. Out of those, 65 studies had to be excluded, because they did not involve pluripotent stem cells at all or their employment was optional. Three studies were excluded, because they had to be withdrawn before enrolling the first participant. Hence, 109 studies remained for further in-depth analysis. Searching the meta-database ICTRP using the same predefined search criteria added 21 additional clinical trials, which were not found in the search using “ClinicalTrials.gov”. The individual search of all databases that are included in the ICTRP did detect one additional relevant clinical trial in the “Australian New Zealand Clinical Trials Registry” (ANZCTR). Altogether, 131 clinical trials involving human pluripotent stem cells were identified and included into further in-depth analyses. For a detailed list of all trials please refer to Supplementary Table 1.

### Global distribution of clinical trials involving pluripotent stem cells

**Fig. 1:**
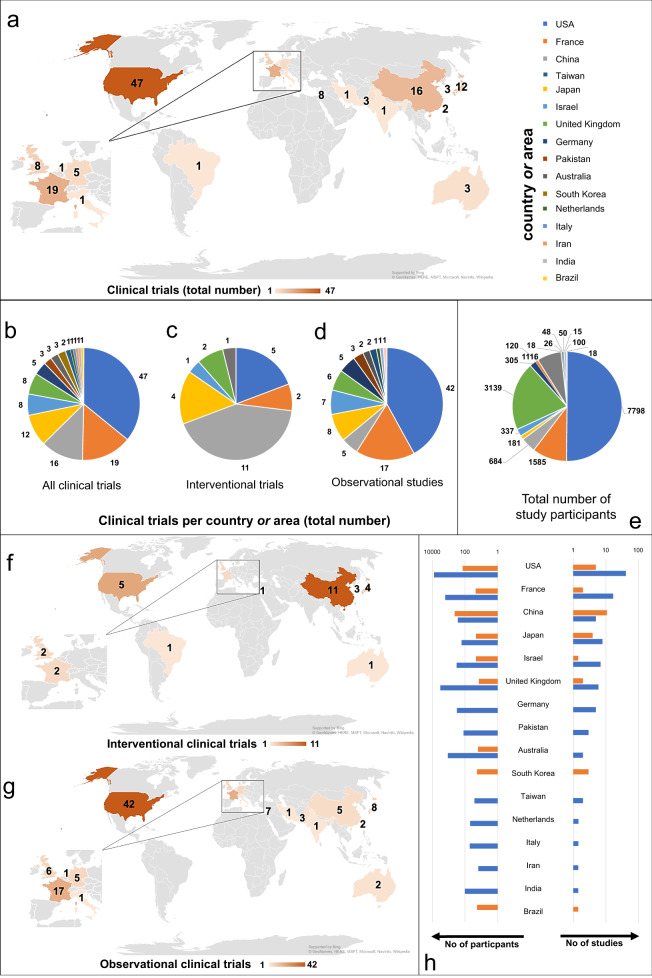
Geographic distribution of studies involving human pluripotent stem cells. **a** Global distribution of all clinical trials involving human pluripotent stem cells. **b** Distribution of all clinical trials involving human pluripotent stem cells. **c** Global distribution of interventional clinical trials involving pluripotent stem cells. **d** Distribution of observational clinical trials involving pluripotent stem cells. **e** Global distribution of all study participants. **f** Global distribution of interventional clinical trials involving pluripotent stem cells. **g** Global distribution of observational clinical trials involving pluripotent stem cells. **h** Comparison between interventional and observational clinical trials and study participants. The horizontal axes have logarithmic scales of different sizes.

All clinical trials were classified according to the country or area they were conducted in. Overall, 36% (47) of all trials were conducted in the USA, 15% (19) in France, 12% (16) in China (entire Chinese territory including Taiwan 14% (18)), 9% (12) in Japan, 6% (8) in the United Kingdom (UK), 6% (8) in Israel, 4% (5) in Germany, 2% (3) in South Korea, 2% (3) in Australia, 2% (3) in Pakistan, 2% (2) in Taiwan, 1% (1) in Brazil, 1% (1) in India, 1% (1) in Italy, 1% (1) in the Netherlands and 1% (1) in Iran (see Fig. 1a, b). The study could be classified as interventional trial in 22.9% (30) and as observational in 77.1% (101). When focusing on interventional studies (characterized by a (re-)transplantation of PSCs into humans), China was by far the leading country with 36.7% (11) of all interventional studies. Also in the USA (16.7%, 5), Japan (13.4%, 4), and South Korea (10.0%, 3) several interventional studies have been performed (Fig. 1c and f). The distribution of observational clinical studies across the globe was as follows: 41.6% (42) of the trials took place in the USA, 16.8% (17) in France, 7.9% (8) in Japan, 5.9% (6) in the UK, 6.9% (7) in Israel, 5.0% (5) in Germany and 5.0% (5) in China (see Fig. 1d and g). These results indicate that, although most studies are performed in the USA, a magnitude of these studies have an observational character. In China much less studies are performed in total, however, with the majority of them being interventional. This trend can also be observed for other Asian countries, such as Japan or South Korea. In total, all studies together comprise 15,540 participants. Interventional studies involved 15.0 (±15.0) participants on average, compared to an average of 149.4 (±389.1) participants in observational studies. It therefore becomes obvious, that most study participants can be found in countries, in which a particularly large number of observational clinical studies were performed. 50.2% (7798) of all participants were included into studies in the USA, followed by 20.2% (3139) in the UK and 10.2% (1585) in France (see Fig. 1e). China, South Korea and Brazil are the only countries in which both, the amount of study participants and the number of studies, is higher for interventional clinical trials than for observational ones. In European countries the numbers of studies and patients of observational trials clearly exceeds the ones of interventional studies. There are even several European countries, where no interventional study involving PSCs was found at all (e.g., Germany, Italy, and the Netherlands; see Fig. 1h). In addition, it could be observed, that in many countries not a single clinical trial involving PSCs is registered. This applies e.g., to all countries on the African continent, Russia, large parts of Europe, and Southern America.

### Target diseases of clinical trials involving pluripotent stem cells

**Fig. 2:**
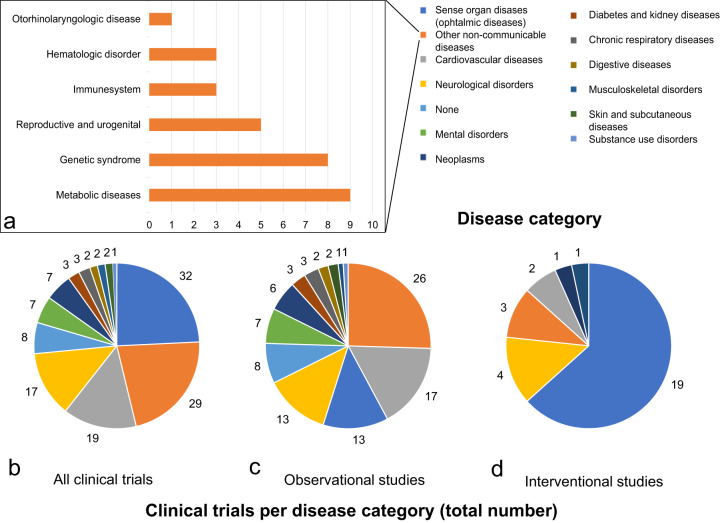
Disease categories targeted in clinical trials involving human pluripotent stem cells. The legend shown on the top right of Fig. 2 is color coordinated with all three pie charts. **a** Sub-classification of the category “non-communicable diseases”. **b** Distribution of clinical trials involving pluripotent stem cells dependent on the targeted disease. **c** Distribution of observational clinical trials dependent on the targeted disease. **d** Distribution of interventional clinical trials dependent on the targeted disease.

PSCs have been investigated regarding the treatment of various diseases affecting several different organ systems. We, therefore, analyzed all clinical trials obtained using the multistep search algorithm regarding the targeted disease. The diseases were classified according to the Global Burden of Disease Study 2017^36^ (Fig. 2a). Sense organ diseases, more precisely ophthalmic diseases, were targeted in the highest number of clinical trials (24.4%, 32) followed by other non-communicable diseases (22.1%, 29), cardiovascular diseases (14.5%, 19) and neurological disorders (13.0%, 17). For a detailed overview please refer to Fig. 2b.

The category “other non-communicable diseases” includes studies focusing on metabolic diseases (6.9% of all studies, 9), genetic syndromes (6.1%, 8), reproductive and urogenital diseases (3.8%, 5), defects of the immune system (2.3%, 3), hematologic disorders (2.3%, 3) and otorhinolaryngologic diseases (0.8%, 1) (Fig. 2a). The category “none” includes studies that are concerned with the assessment of reprogramming processes (2.3% of all studies, 3) the exploration of differentiation mechanisms (2.3%, 3) and cell banking (1.5%, 2). Analysis of the different disease categories involved in observational type clinical trials revealed that mainly the following diseases were targeted: Other non-communicable diseases (25.7%, 26), cardiovascular diseases (16.8%, 17), neurological disorders (12.9%, 13) and sense organ diseases (12.9%, 13) (Fig. 2c). Interventional clinical trials focused on sense organ diseases (63.3%, 19), holding a big lead over neurological disorders (13.3%, 4), other non-communicable diseases (10%, 3), cardiovascular diseases (6.7%, 2), neoplasms (3.3%, 1) and musculoskeletal disorders (3.3%, 1) (Fig. 2d). In summary, ophthalmic diseases are in the main focus of a major part of all clinical trials involving human PSCs, especially regarding interventional studies. If the categories including ophthalmic diseases and neurological disorders are combined, this group represents the majority of interventional PSC clinical trials (76.6%, 23). Trials focusing on ophthalmic diseases, neurological disorders and cardiovascular diseases account for 51.9% (68) of all PSC – studies.

### Study duration of clinical trials

**Fig. 3:**
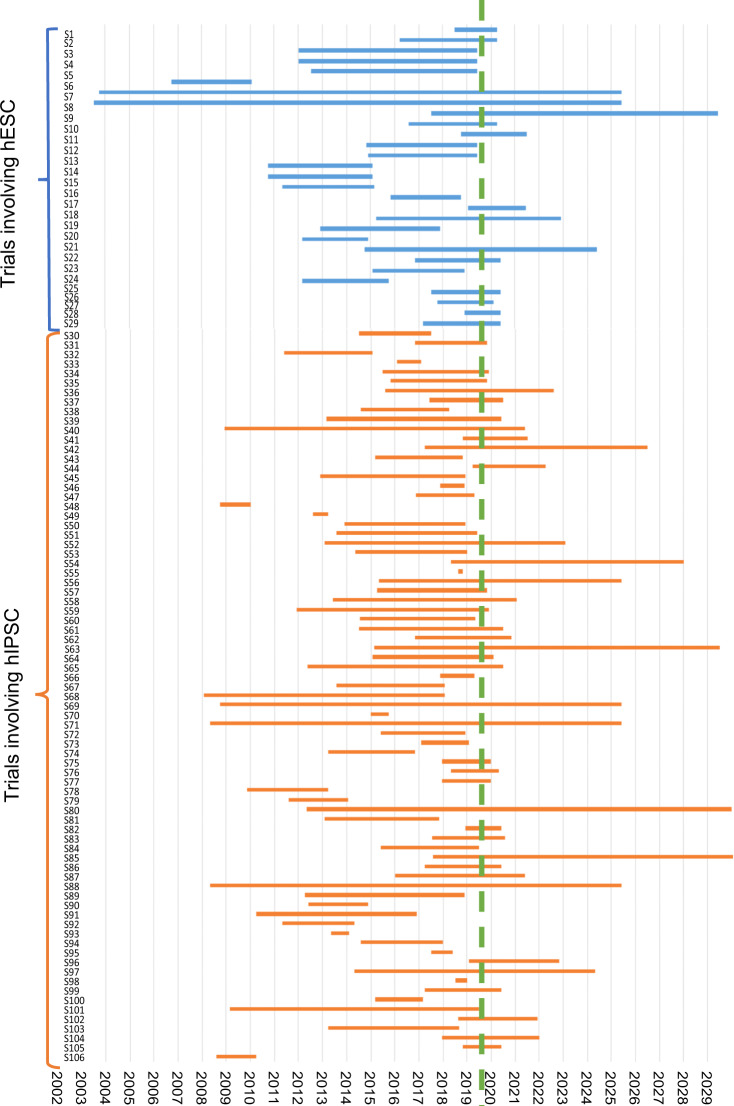
Comparison of starting point and study duration dependent on the use of either one of the two stem cell types, hiPSC and hESC. The blue bars show the starting point and study duration of clinical studies involving hESCs, the orange bars depict the same information for studies involving hiPSC. The green dashed line shows the time of data collection. The *x* axis shows the time in years, the *y* axis shows the study numbers. A list of the respective studies can be found in Supplementary Table 1.

**Fig. 4:**
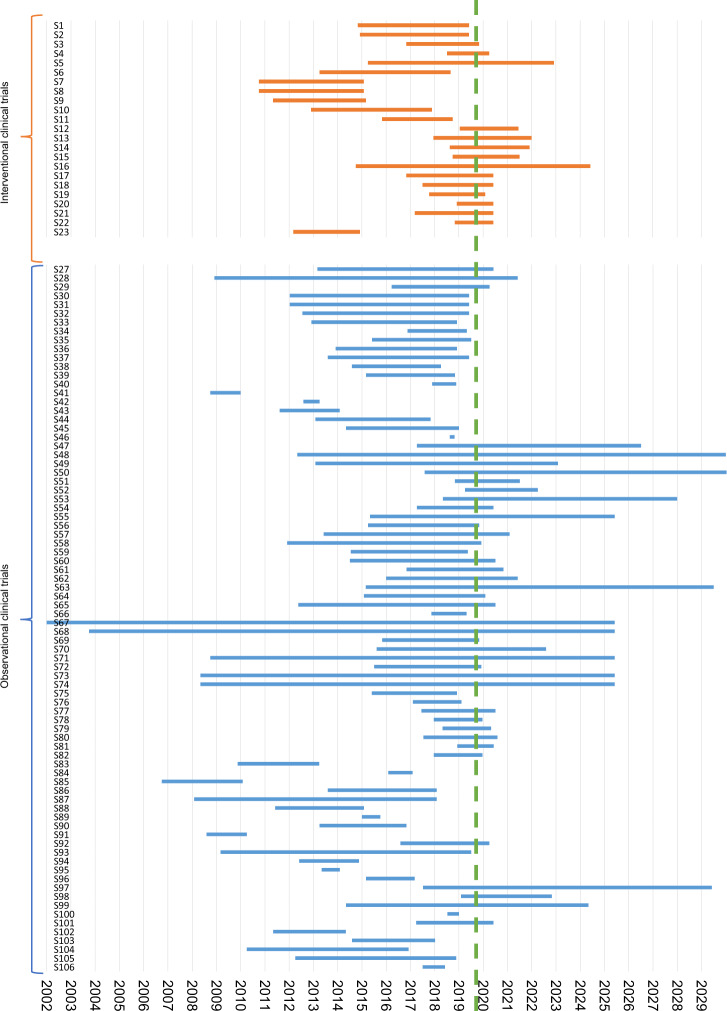
Comparison of starting point and (scheduled) study duration dependent on the study design. The blue bars show the starting point and study duration of observational clinical studies, the orange bars depict the same information for interventional clinical trials. The green dashed line shows the time of data collection. The *x* axis shows the time in years, the *y* axis shows the study numbers. A list of the respective studies can be found in Supplementary Table 1.

The study duration of clinical trials involving PSCs was analyzed according to the information available from the different databases. The mean duration of trials involving hESCs was 5.8 (±5.1) years compared to 5.2 (±4.2) years for studies using hiPSCs (*p* = 0.635; see Fig. 3 and Supplementary Fig. 1). Clinical trials of the interventional type were significantly shorter (3.8 years, ±1.8) compared to studies of the observational type (5.9 years (±5.0), *p* = 0.002, see Fig. 4 and Supplementary Fig. 2). However, only 106 out of 131 trials could be considered, since the end date was not mentioned in 25 (19.1%) trials.

### Type of study and type of stem cells used

**Fig. 5:**
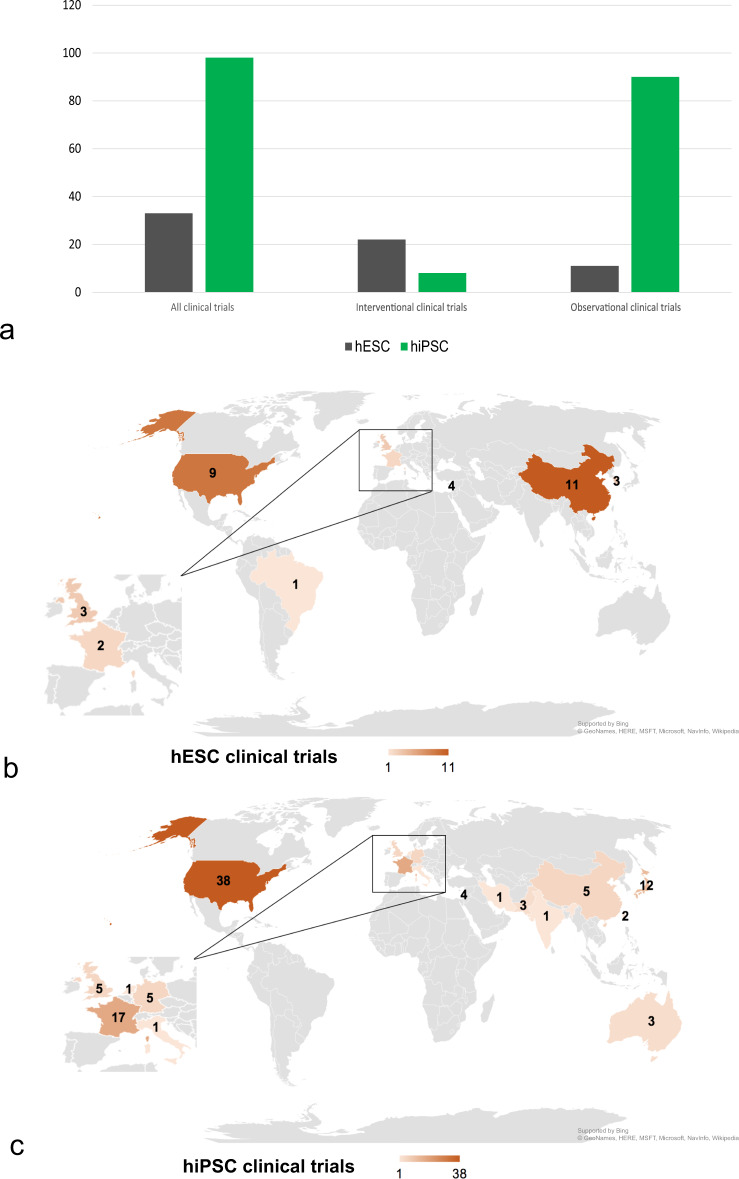
Usage of pluripotent stem cell types (hESC and iPSC) dependent on geographic distribution and study design. **a** Distribution of all clinical trials and interventional and observational trials respectively, dependent on the usage of hESC or hiPSC. **b** Choropleth map presenting the geographic distribution of all clinical trials involving hESCs. **c** Choropleth map presenting the geographic distribution of all clinical trials involving hiPSCs.

77.1% (101) of all clinical trials involving human PSCs were observational, while 22.9% (30) were interventional. In 73.3% (22) of interventional studies hESCs were used, while iPSCs were involved in only 26.7% (8). The origin of the cells was allogeneic in 93.3% (28) and autologous in 6.7% (2). However, when it comes to observational clinical trials, the frequency of the used stem cell type is vice versa. In these studies, hESCs were used in only 10.9% (11), while iPSCs were employed in 89.1% (90). The distribution of all clinical trials dependent on the characteristics interventional/observational and hESC/iPSC is summarized in Fig. 5a. In-depth analysis of the geographical distribution of clinical trials using different types of hPSCs revealed that most studies involving hESCs were conducted in China (33.3%; 11), followed by the USA (27.3%, 9), Israel (12.2%, 4), the UK (9.1%, 3), and South Korea (9.1%, 3) (see Fig. 5b). Concerning clinical trials involving hiPSC, 38.4 % (38) were performed in the USA, 17.2% (17) in France, and 12.1% (12) in Japan (see Fig. 5c).

### Study design and clinical study phase of interventional trials

All interventional studies were open label, non-randomized (100.0%, 30). 90.0% (27) were carried out in one center and 10.0% (3) were multi-center studies. The studies comprised one single arm in 43.3% (14), in 40% (13) the trials enclosed two or more intervention groups, each receiving treatment with pluripotent stem cells (or cells derived from pluripotent stem cells), 6.7% (2) of the studies included a group without intervention and 3.3% (1) a historical control.

In 30% (9) the trial was categorized as Phase I study by the authors, in 50.0% (15) as Phase I/II, in 6.7% (2) the authors declared that this categorization is not applicable and in 13.3% (4) no information regarding the clinical study phase was given. For details on interventional studies please refer to Table 1 and Supplementary Table 2.

**Table 1:** Characteristics of interventional clinical trials involving pluripotent stem cells.

ID number	Title	Cell type	Origin	Target disease	Country *or* area	No. patients enrolled	Intervention	Status (2019)
NCT03222453	Thalassemia treatment based on the stem cell technology	hiPSC	Autologous	Beta-thalassemia	China	2	Injection of hematopoietic stem cells	Results submitted to ClinicalTrials.gov
NCT01674829	A Phase I/IIa, open-label, single-center, prospective study to determine the safety and tolerability of sub-retinal transplantation of human embryonic stem cell-derived retinal pigmented epithelial(MA09-hRPE) cells in patients with advanced dry age-related macular degeneration	hESC-derived RPE; MA09-hRPE	Allogeneic	Dry age-related macular degeneration	South Korea	12	Sub-retinal Transplantation	Active, not recruiting
NCT02903576	Stem cell therapy for outer retinal degenerations	hESC-derived RPE	Allogeneic	Age-related macular degeneration, Stargardt’s disease or exudative age-related macular degeneration	Brazil	18	Injection of hESC-RPE in suspension versus injection of hESC-RPE seeded in a substrate	Not yet reported, recruitment status unknown
NCT01625559	Safety and tolerability of MA09-hRPE cells in patients with Stargardt’s macular dystrophy (SMD)	hESC-derived RPE cells (MA09-hRPE)	Allogeneic	Stargardt’s macular dystrophy	South Korea	3	Sub-retinal transplantation	Not yet reported, recruitment status unknown
NCT03763136	Treating heart failure with hPSC-CMs	hiPSCs- derived cardiomyocytes	Allogeneic	Heart failure	China	5	Injection into the myocardium	Recruiting
NCT03046407	Treatment of dry age-related macular degeneration disease with retinal pigment epithelium-derived from human embryonic stem cells	hESC-derived RPE	Allogeneic	Dry age-related macular degeneration	China	10	Subretinal transplantation	Recruiting
NCT03944239	Safety and efficacy of subretinal transplantation of clinical human embryonic stem cell-derived retinal pigment epitheliums in treatment of retinitis pigmentosa	hESC-derived RPE	Allogeneic	Retinitis pigmentosa	China	10	Subretinal transplantation	Recruiting
NCT03482050	Study to evaluate transplantation of astrocytes derived from human embryonic stem cells, in patients with amyotrophic lateral sclerosis	hESC-derived astrocytes	Allogeneic	Amyotrophic lateral sclerosis	Israel	21	intrathecal (spinal) injection	Recruiting
NCT02755428	Subretinal transplantation of retinal pigment epitheliums in treatment of age-related macular degeneration diseases	hESC-derived RPE	Allogeneic	Dry age-related macular degeneration	China	10	Subretinal transplantation	Recruiting
NCT03119636	Safety and efficacy study of human ESC-derived neural precursor cells in the treatment of Parkinson’s disease	hESC- derived neural precursor cells	Allogeneic	Parkinson’s disease	China	50	Stereotaxic intra-striatal injection	Recruiting
NCT02286089	Safety and efficacy study of opregen for treatment of advanced dry-form age-related macular degeneration	hESC-derived RPE	Allogeneic	Age-related macular degeneration	USA + Israel	24	Subretinal transplantation	Recruiting
NCT03877471	Mesenchymal stem cells - like cell transplantation in women with primary ovarian insufficiency	hESC-derived MSC-like cells	Allogeneic	Primary ovarian insufficiency	China	28	Injection into ovaries bilaterally	Recruiting
NCT03841110	FT500 as monotherapy and in combination with immune checkpoint inhibitors in subjects with advanced solid tumors	iPSC-derived NK cell cancer immunotherapy	Allogeneic	Advanced solid tumors	USA	76		Recruiting
JPRN-UMIN000032989	Clinical trial of human (allogeneic) induced pluripotent stem cell-derived cardiomyocyte sheet for severe cardiomyopathy	iPS cell-derived cardiomyocytes	Allogeneic	Ischemic cardiomyopathy	Japan	3	iPS cell-derived cardiomyocyte sheet transplantation	Recruitment complete
NCT03963154	Interventional study of implantation of hESC-derived RPE in patients With RP due to monogenic mutation	hESC-derived RPE	Allogeneic	Retinitis pigmentosa due to monogenic mutation	France	12	single central subretinal implantation of a hESC-derived RPE monolayer in one eye	Recruiting
NCT03305029	The safety and tolerability of sub-retinal transplantation of SCNT-hES-RPE cells in patients with advanced Dry AMD	SCNT hESC-derived RPE	Allogeneic	Dry age-related macular degeneration	South Korea	3	Subretinal transplantation	Recruitment status unknown
NCT02057900	Transplantation of human embryonic stem cell-derived progenitors in severe heart failure (ESCORT)	hESC- derived CD15+Isl-1+ progenitors	Allogeneic	Ischemic heart disease	France	10	fibrin gel embedding hESC-derived CD15+Isl-1+ progenitors	Completed, results published^18^
NCT01469832	Safety and tolerability of sub-retinal transplantation of human embryonic stem cell-derived retinal pigmented epithelial (hESC-RPE) cells in patients with Stargardt’s Macular Dystrophy (SMD)	hESC-derived RPE cells (MA09-hRPE)	Allogeneic	Stargardt’s macular dystrophy	UK	12	Subretinal injection	Completed, results published^61^
NCT01345006	Sub-retinal transplantation of hESC-derived RPE(MA09-hRPE) cells in patients with Stargardt’s Macular Dystrophy	hESC-derived RPE cells (MA09-hRPE)	Allogeneic	Stargardt’s macular dystrophy	USA	13	Subretinal injection	Completed, results published^5,62^
NCT01344993	Safety and tolerability of sub-retinal transplantation of hESC-derived RPE (MA09-hRPE) cells in patients with advanced dry age-related macular degeneration	hESC-derived RPE cells (MA09-hRPE)	Allogeneic	Dry age-related macular degeneration	USA	13	Subretinal injection	Completed, results published^5,62^
JPRN-UMIN000011929	A Study of transplantation of autologous induced pluripotent stem cell (iPSC) derived retinal pigment epithelium (RPE) cell sheet in subjects with exudative age-related macular degeneration	hiPSC-derived RPE	Autologous	Exudative age-related macular degeneration	Japan	6	Transplantation of iPSC-derived RPE cell sheet	Complete, results published^19^
NCT02590692	Study of subretinal implantation of human embryonic stem cell-derived RPE cells in advanced Dry AMD	hESC-derived RPE	Allogeneic	Dry age-related macular degeneration	USA	16	Subretinal implantation	Active, not recruiting
NCT03839238	Safety observation on hESC-derived MSC like cell for the meniscus injury	hESC- derived MSC like cell	Allogeneic	Meniscus injury	China	18	hESC- derived MSC like cells	Active, not recruiting
NCT02923375	A study of CYP-001 for the treatment of steroid-resistant acute graft versus host disease	iPSC- derived MSCs	Allogeneic	Graft vs host disease	Australia + UK	16	IV infusion on two occasions	Active, not recruiting
NCT01691261	A study of implantation of retinal pigment epithelium in subjects with acute wet age-related macular degeneration	hESC-derived RPE	Allogeneic	Age-related macular degeneration	UK	2	Implantation of RPE monolayer immobilized on a polyester membrane.	Active, not recruiting
NCT02749734	Clinical study of subretinal transplantation of human embryo stem cell-derived retinal pigment epitheliums in treatment of macular degeneration diseases	hESC-derived RPE	Allogeneic	Macular degeneration Stargardt’s macular dystrophy	China	15	Subretinal transplantation	Recruitment status unknown
JPRN-UMIN000033564	Kyoto trial to evaluate the safety and efficacy of iPSC-derived dopaminergic progenitors in the treatment of Parkinson’s disease	hiPSC-derived dopaminergic progenitors		Parkinson’s disease	Japan	7	Transplantation into the corpus striatum	Recruitment suspended
JPRN-UMIN000026003	A Study of transplantation of allogenic induced pluripotent stem cell (iPSC) derived retinal pigment epithelium (RPE) cell suspension in subjects with neovascular age-related macular degeneration	iPSC-derived RPE	Allogeneic	Neovascular age-related macular degeneration	Japan	5	Subretinal transplantation	Recruitment complete; follow-up continuing
ChiCTR-OCB-15007054	Clinical study of subretinal transplantation of clinical human embryonic stem cells derived retinal pigment epitheliums in treatment of dry age-related macular degeneration diseases	hESC-derived RPE	Allogeneic	Dry age-related macular degeneration	China	10	Subretinal transplantation	Recruiting
ChiCTR-OCB-15005968	The clinical trial of human embryonic stem cell-derived epithelial cells transplantation in the treatment of severe ocular surface diseases	hESC-derived epithelial cells	Allogeneic	Severe ocular surface disease	China	20	Corneal-epithelium-like-cell transplantation	Recruiting

*hESC* human embryonic stem cells, *hiPSC* human induced pluripotent stem cell, *RPE* retinal pigment epithelium, *hRPE* human retinal pigment epithelium, *MSC* mesenchymal stem cell, *UK* United Kingdom.

## Discussion

Human pluripotent stem cells hold great potential for novel therapeutic approaches to regenerate or replace functionally impaired tissues^20,37–40^. In addition, the use of autologous cells for in vitro disease modeling and drug testing without re-transplantation into the host has also become an increasingly important scientific branch of regenerative medicine^41,42^. Following the initial report by Yamanaka and Takahashi in 2006^9^, the number of scientific publications on pluripotent stem cells has virtually exploded over the past years. Consequently, it can be assumed that also the number of clinical trials, in the sense of studies on PSCs involving human subjects, may have been increasing in parallel. While scientific publications represent an indicator for the scientific interest in a certain technology, the number of clinical trials indicates how many attempts have been made to actually translate the technology from the bench to the clinical phase as it represents the final stage prior to a potential standard use in human patients. However, the published information contained in research studies is generally limited by a significant publication bias, implying that negative results may not be published at all due to rejection by editors or reviewers, competing interests or lack of interest to publish^43,44^. In addition, results may not be published due to a lack of interest by the sponsoring institution. Therefore, we have focused on the comprehensive analysis of the past and present status of clinical trials on pluripotent stem cells to generate an overview on the actual number and content of clinical trials involving this promising technology.

So far, several studies have been focusing on the trends in clinical trials involving “stem cells” in general. Monsarrat et al. published an analysis of clinical trials involving stem cells listed on “ClinicalTrials.gov”. In this work he assessed the different organ systems and diseases involved in these trials, including comparisons between different study phases and stem cell types^31^. Squillaro et al. focused on clinical trials involving “mesenchymal stem cells”, investigating different clinical findings and therapeutic effects^33^. Fung et al. analyzed to which extent authors of clinical trials involving stem cells had already published their results by using PubMed, Embase and Google Scholar^30^. However, so far, there is no comprehensive, systematic analysis addressing the geographic distribution, concerned organ systems and used cell types of clinical trials on human pluripotent stem cells. In addition, these previous studies were either limited regarding the type of stem cell used, e.g., focusing on “multipotent stem cells” only^33^, and/or their search was only conducted in one database (mostly “ClinicalTrials.gov”)^30–33^. Even though “ClinicalTrials.gov” is the largest database for clinical trials and represented in more than 200 countries around the globe, a search query in this database does not guarantee completeness. Alternative meta-databases collecting data from even more countries and databases, such as the ICTRP database from the WHO, have not been included in several studies^45^, which implies that some trials may have been missed.

Therefore, in the present analysis, we complemented our systematic search on “ClinicalTrials.gov” by using the ICTRP database of the WHO as well as by performing a search at each individual platform that is included in the ICTRP repository. This multi-step approach should enable the most complete overview on clinical trials involving “pluripotent stem cells”. Following a stringent inclusion/exclusion procedure, 131 studies remained that could be classified as clinical trials involving PSCs. The magnitude of these studies (77.1%) was of observational nature, which means that no cells were transplanted into patients for therapeutic use. These studies mainly included trials where cells were isolated from patients or volunteers for the subsequent use as part of reprogramming experiments. This also implies that only a minority of studies (22.9%) were of an interventional study type, where cells were actually transplanted into patients for therapeutic purposes. From the view point of translational medicine, these interventional studies are of greater interest, since the introduction of the iPSC technology was associated with a significant hope for future clinical use in the sense of cellular transplantation^8,46–49^. While the total number of clinical trials involving iPSCs (74.8%) was substantially higher than the ones involving ESCs (25.2%), the picture changes completely when focusing on interventional studies. Here, in the majority (73.3%) of trials ESCs were involved and only in few studies (26.7%) iPSCs were used for the intervention. However, when considering the increasing number of trials on iPSCs, a future switch towards interventional iPSC trials might just be a matter of time. In particular the introduction of non-integrating transgene-free reprogramming approaches may further support the clinical translation of the iPSC technology as the issue of in situ carcinogenicity of iPSCs due to remaining viral particles may be solved by these technologies^50^.

Also, when focusing on the geographical regions, where the studies have been performed, it becomes obvious that there are substantial differences between different study types. While regarding observational studies the greatest part of all trials was performed in the USA (41.6%) and in France (16.8%), the majority of interventional studies was performed in Asian countries (China 36.7%, Japan 13.3%, South Korea 10.0%). The major reasons for this may be seen in the differences in legal and/or regulatory restrictions on ESC research in Western countries, in different research policies as well as in different national ethical regulations of different countries concerning pluripotent stem cell research^7,51–54^. In many countries of the European region, the harvest of stem cells from a human embryo for research purposes is prohibited, however, in some countries research can be conducted using imported pluripotent cells^55^. In the USA, stem cell research is regulated by National Academies (NAS) Guidelines, which allows the derivation of ESC lines from human embryos, however, additionally, some US states have adopted their own regulations^56^. In China, where research on human embryos and cloning for therapeutic purposes is in general permitted, state regulations established in 2015 attempt to limit clinical trials using stem cells to research centers^57,58^. Other potential factors might be the differences in the focus of research institutions on a particular topic, the policies of funding agencies as well as the societal perceptions on pluripotent stem cell research in general^59^.

When focusing on the targeted diseases of PSC clinical trials, it becomes evident that “sense organ diseases”, more precisely ophthalmic diseases, are targeted in 24.4% (32) of all studies. Regarding the group of interventional studies even 63.3% of all trials addressed the eyes. The majority of observational studies was concerned with other non-communicable diseases (25.7%, 26), cardiovascular diseases (16.8%, 17), neurological disorders (12.9%, 13), and ophthalmic diseases (12.9%, 13). In particular the relatively high proportion of - especially interventional - clinical trials in the field of ophthalmology is striking. A major reason for this may not only lie in the different ocular pathologies that profit from cellular replacement, it may also be due to anatomic circumstances, since the eye is easily accessible for stem cell transplantation and in the case of adverse events life-threatening complications are scarce^4,60^. This lack of fatal complications represents a major advantage over diseases concerning other organ systems, such as cardiovascular diseases, when introducing a new therapeutic concept. However, also the pathophysiology of targeted ophthalmologic diseases forms the basis for the broader clinical investigation of hPSCs in this field. Ophthalmologic diseases, such as Stargardt’s macular dystrophy or atrophic age-related macular degeneration, are predominantly treated as part of these clinical trials^5,19^. These diseases share the pathophysiologic element of progressive cellular degeneration that may be treated by stem cells replacing the impaired tissues^5^. In the end, it is the combination of all the reasons mentioned above that may form the basis for the disproportionally high fraction of PSC interventional clinical trials focusing on ophthalmologic diseases^4,60^.

Interestingly, an in-depth analysis of the group of interventional studies also revealed several limitations of these recent clinical trials on pluripotent stem cells. This includes a lack of placebo control groups (0%) or untreated control groups (6.7%), a lack of long-term follow-up periods (only one study with published results had a follow-up of >12 months) and a lack of reporting (only 27.8% of all interventional trials without active recruitment have already been published in a peer-reviewed journal). A major limitation of the present analysis, as well as of all studies focusing on the analysis of clinical trial databases in general, might be a reporting bias. In particular regarding observational studies, it has to be assumed that not all studies may have been registered on public databases, limiting the representativeness of the data on this type of trials.

In summary, the present analysis shows that, although a large series of clinical trials involving PSCs has been registered in public databases, only a small part has been focusing on the actual transplantation of cells. The greatest part of these interventional studies was focusing on the use of ESCs in the field of ophthalmology. Therefore, in spite of the “hype” on iPSC research in recent years also fostered by the Nobel Prize in 2012, the iPSC technology has not reached the clinical investigational phase on a broader scale yet. The future will tell us, if the iPSC technology will ultimately overcome the challenges associated with their clinical use - such as carcinogenicity, lack of in situ integration^49,61^, genomic instability^27^, immunological rejection^28^ or lack of quality control criteria^62^ - and whether it will finally make its way into routine clinical use.

## Methods

### Systematic search of databases

The aim of the study was to perform a comprehensive analysis of all clinical trials involving pluripotent stem cells based on a defined sequence of database searches. First a search was performed in the database “ClinicalTrials.gov” using the search terms “pluripotent” OR “pluripotent stem cell” OR “induced pluripotent stem cell” OR “iPSC” OR “embryonic stem cell”. In parallel a search was performed on the “International Clinical Trials Registry Platform” (ICTRP) using the identical search terms as mentioned above. This primary search was performed in August 2019. The search algorithm is summarized in Supplementary Fig. 3. In total 177 studies were identified at “ClinicalTrials.gov”. Among them, 68 clinical studies had to be excluded because 65 of them did not involve pluripotent stem cells or the employment of pluripotent stem cells was optional. The three remaining studies were excluded since they were withdrawn before enrolling its first participant. This search was complimented by a search on the ICTRP portal site, which is provided by the WHO. This database combines several different national and international databases. These individual databases were searched separately in order to maximize the number of relevant trials identified. Based on the ICTRP portal site 21 additional clinical trials could be added to the final analysis after excluding duplicated search results also obtained from “ClinicalTrials.gov”. The individual search of databases included in the ICTRP-based meta search did only detect one additional relevant clinical trial in the “Australian New Zealand Clinical Trials Registry” (ANZCTR). In total, 131 appropriate clinical trials involving pluripotent stem cells were identified, downloaded and consistently analyzed using Microsoft Excel (Microsoft Inc., USA). The world maps were created using Microsoft Excel (Microsoft Inc., USA). Microsoft product screen shot(s) of the world maps were reprinted with permission from Microsoft Corporation. The following properties of the 131 studies have been content of the analysis: “Registration number” (NCT number, if possible), “study title”, “recruitment status”, “study type” (observational or interventional), “type of stem cell” (hESC or hiPSC), “study target”, “target disease”, “corresponding organ system”, “sponsor”, “location”,”study start”, “estimated study completion” and “number enrolled”.

### Content analysis of clinical trials

The studies were generally classified as observational or interventional study types by the study authors. However, in some cases the “interventional” type of the study mentioned on the database was derived from an “invasive” cell harvesting procedure only, such as a blood draw or a skin biopsy. In our analysis the term “interventional” study type was uniformly reserved for studies, in which cells were (re)transplanted into a patient using an interventional procedure by itself. An invasive cell harvesting procedure, such as a blood draw, was not sufficient to fulfill this criterion. Accordingly, a clinical trial was assigned to the “observational” study type, if the intervention only served to obtain primary cells. Based on these considerations, 20 apparently “interventional” clinical trials were re-categorized as “observational” studies. In two supposedly “observational” clinical trials it was the other way round, they were re-categorized as “interventional” clinical trials. The search for clinical trials and parts of the results section were generated as part of a medical diploma thesis of one of the first authors of this publication.

### Biostatistical analysis

All data are presented in percentage (and absolute numbers). The study duration of different study types was compared using an unpaired Student’s *t* test. *P* values < 0.05 were considered statistically significant. All analyses were performed using either Microsoft Excel (Microsoft Inc., USA) or SPSS 24.0 (SPSS Inc., USA). Individual sub-groups were compared graphically in the form of pie or bar charts. In addition, choropleth maps, generated using Microsoft Excel (Microsoft Inc., USA), were used to indicate the geographical distribution of the involved studies.

### Reporting summary

Further information on research design is available in the Nature Research Reporting Summary linked to this article.

## Supplementary information

Supplementary Information

Reporting Summary

## Data Availability

The data contained in this manuscript was derived from the following publicly available databases: “ClinicalTrials.gov” and the ICTRP database of the WHO (and its individual databases).
