# Supplementary material for: Global trends in clinical trials involving pluripotent stem cells: a systematic multi-database analysis
Source: NPJ Regen Med. 2020 Sep 11;5:15. doi: 10.1038/s41536-020-00100-4 (PMC7486930; doi:10.1038/s41536-020-00100-4)
Supplement: Supplementary file 1 — Supplementary Information [file 41536_2020_100_MOESM1_ESM.pdf]

Supplementary Material

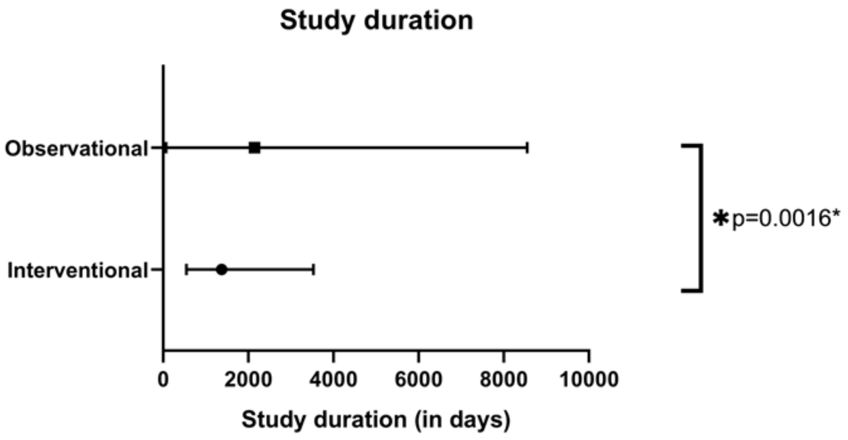

Study group	Interventional trials	Observational trials
Number of values	26	80
Mean	1373.5	2147.3
Std. Deviation	650.8	1890.9
Std. Error of Mean	127.6	202.3

*unpaired, two-tailed t-test

Supplementary Figure 1. Statistical analysis of the study duration of observational versus interventional studies.

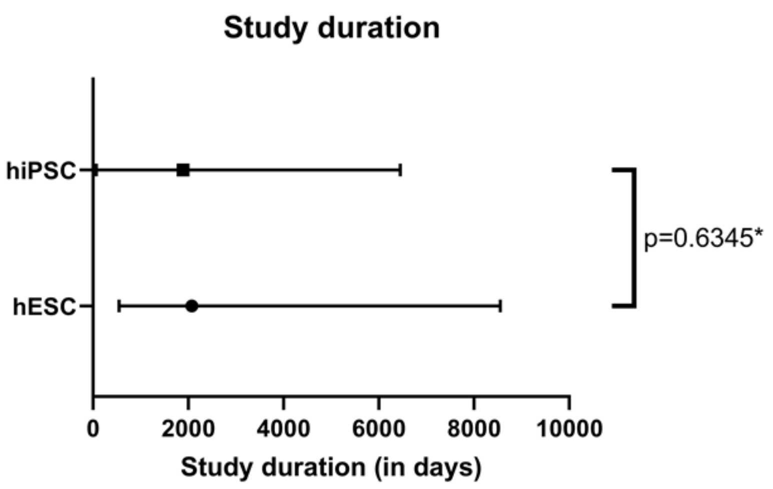

Study group	hESC	hiPSC
Number of values	30	79
Mean	2073.1	1889.5
Std. Deviation	1882.5	1515.0
Std. Error of Mean	343.7	170.5

*unpaired, two-tailed t-test

Supplementary Figure 2. Statistical analysis of the study duration of clinical trials involving hiPSCs versus hESCs.

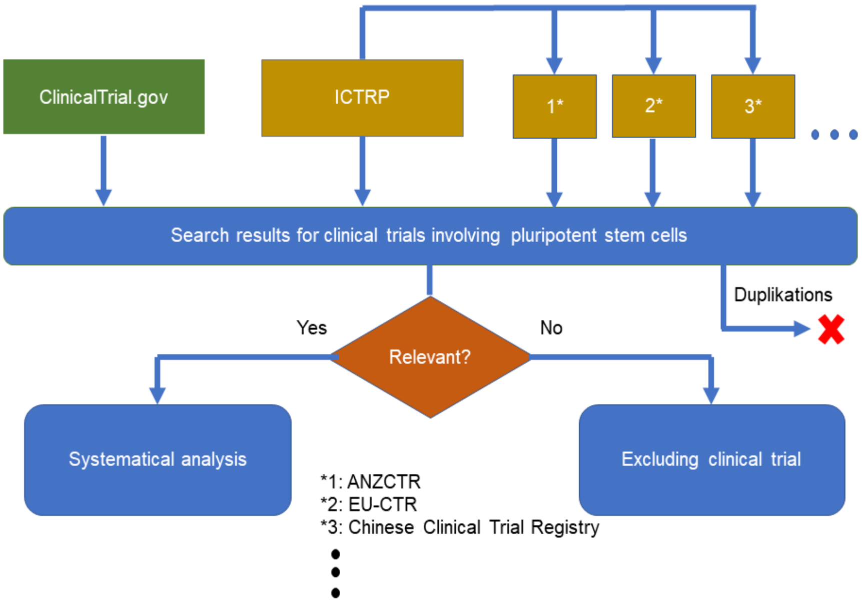

Supplementary Figure 3. Algorithm for systematic multi-database search on clinical trials involving pluripotent stem cells.

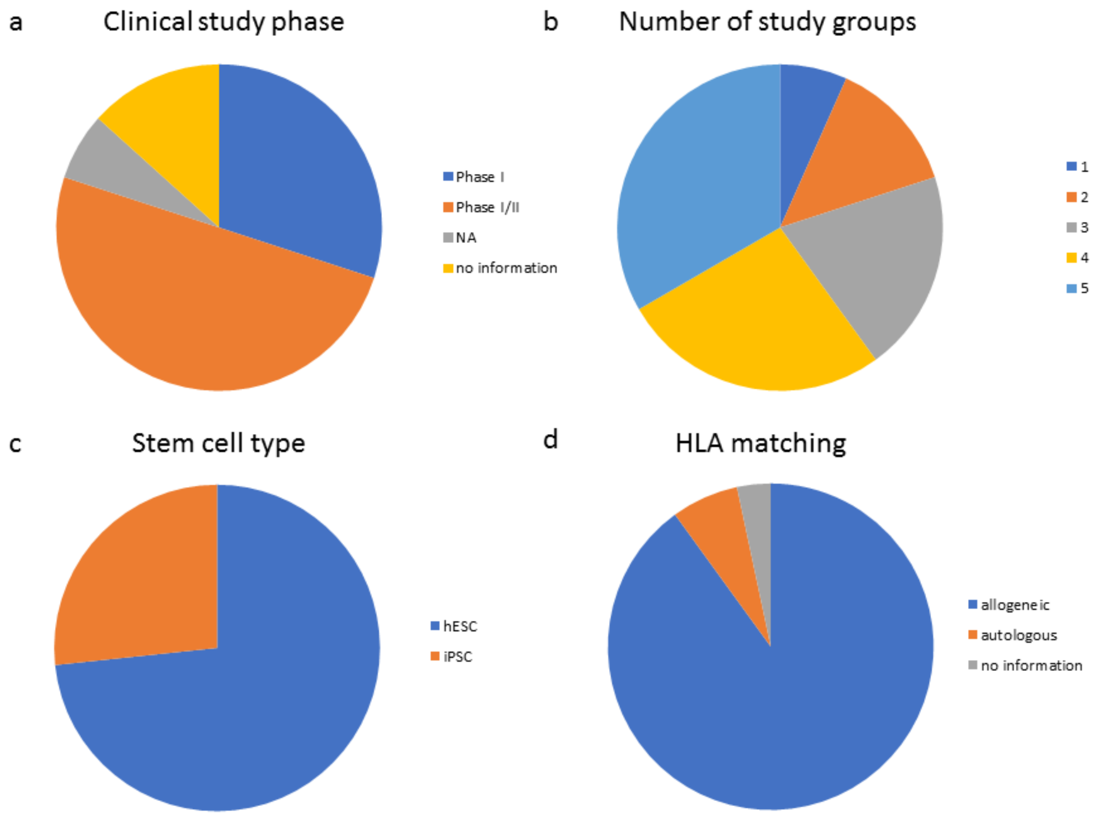

Supplementary Figure 4. Characteristics of interventional studies

Number	Database	Study title	NCT number or ID number
1	ClinicalTrials.gov	Induced Pluripotent Stem Cells for Niemann Pick Disease	NCT03883750
2	ClinicalTrials.gov	Organoids Derived From Induced-Pluripotent Stem Cells (iPS) From Patients With High Grade Astrocytoma (GLIOMANOID)	NCT03971812
3	ClinicalTrials.gov	Modeling and Pharmacological Targeting of Genetic Cardiomyopathy in Children Via Cardiomyocytes Derived From Induced Pluripotent Stem Cells (DMDstem) (DMDstem)	NCT03696628
4	ClinicalTrials.gov	Human iPSC for Repair of Vasodegenerative Vessels in Diabetic Retinopathy (iPSC)	NCT03403699
5	ClinicalTrials.gov	Establishment of Human Cellular Disease Models for Morquio Disease (IPSMORQUIO)	NCT03872713
6	ClinicalTrials.gov	Establishment of Human Cellular Disease Models for Wilson Disease (IPSWILSON)	NCT03867526
7	ClinicalTrials.gov	Generation of Marfan Syndrome and Fontan Cardiovascular Models Using Patient-specific Induced Pluripotent Stem Cells	NCT02815072
8	ClinicalTrials.gov	ASD-specific Induced Pluripotent Stem Cells for Disease Modeling	NCT02720939
9	ClinicalTrials.gov	Treating Heart Failure With hPSC-CMs (HEAL-CHF)	NCT03763136
10	ClinicalTrials.gov	Investigating Cardiovascular Adverse Events Related to Cancer Treatment (InvestiCAT)	NCT03199300
11	ClinicalTrials.gov	Multi-Omics and IPSCs to Improve Diagnosis of Rare Intellectual Disabilities (MIDRID)	NCT03635294
12	ClinicalTrials.gov	Modeling Bronchial Epithelium Modifications Associated With COPD Using iPS (INVECCO)	NCT03181204
13	ClinicalTrials.gov	Thalassemia Treatment Based on the Stem Cell Technology	NCT03222453
14	ClinicalTrials.gov	Stem Cells in NF1 Patients With Tumors of the Central Nervous System	NCT03332030
15	ClinicalTrials.gov	Biomolecular Messages Associated With the Differentiation of Human Induced Pluripotent Stem Cells to Skeletal Muscle Progenitor Cells	NCT02836145
16	ClinicalTrials.gov	Derivation of Induced Pluripotent Stem Cells From an Existing Collection of Human Somatic Cells	NCT00801333

17	ClinicalTrials.gov	Use of Existing Fibroblast Cells to Convert to Induced Pluripotent Stem Cells	NCT00801372
18	ClinicalTrials.gov	Production of iPSC Derived RPE Cells for Transplantation in AMD	NCT02464956
19	ClinicalTrials.gov	Regenerative Cellular Therapies, Physiology, Pathology and Developmental Biology (RCT)	NCT02469207
20	ClinicalTrials.gov	Development of iPS From Donated Somatic Cells of Patients With Neurological Diseases	NCT00874783
21	ClinicalTrials.gov	iPSC Neurons From Adult Survivors of Childhood Cancer Who Have Persistent Vincristine-Induced Neuropathy	NCT02564484
22	ClinicalTrials.gov	Generation of Haploid Stem Cells From Human Germ Cells	NCT01454765
23	ClinicalTrials.gov	Generation of Heart Muscle Cells From Blood or Skin Cells of Breast Cancer Patients	NCT02772367
24	ClinicalTrials.gov	iPS Cells of Patients for Models of Retinal Dystrophies (RETIPS)	NCT03853252
25	ClinicalTrials.gov	Symdeko in Cystic Fibrosis Patients	NCT03506061
26	ClinicalTrials.gov	Treatment of Dry Age Related Macular Degeneration Disease With Retinal Pigment Epithelium Derived From Human Embryonic Stem Cells	NCT03046407
27	ClinicalTrials.gov	Safety and Efficacy of Subretinal Transplantation of Clinical Human Embryonic Stem Cell Derived Retinal Pigment Epitheliums in Treatment of Retinitis Pigmentosa	NCT03944239
28	ClinicalTrials.gov	A Study to Evaluate Transplantation of Astrocytes Derived From Human Embryonic Stem Cells, in Patients With Amyotrophic Lateral Sclerosis (ALS)	NCT03482050
29	ClinicalTrials.gov	Susceptibility Genes in Autism Spectrum Disorders	NCT02628808
30	ClinicalTrials.gov	The Derivation of Human Embryonic Stem Cell Lines From PGD Embryos	NCT00353210
31	ClinicalTrials.gov	Subretinal Transplantation of Retinal Pigment Epitheliums in Treatment of Age-related Macular Degeneration Diseases	NCT02755428
32	ClinicalTrials.gov	Derivation of New Human Embryonic Stem Cell Lines Lines for Clinical Use	NCT00353197
33	ClinicalTrials.gov	Biological Collection of Kidney Cells (CRHiPS)	NCT03364504
34	ClinicalTrials.gov	Cell Collection to Study Eye Diseases	NCT01432847

35	ClinicalTrials.gov	Generation of a Cellular Model of CADASIL From Skin Fibroblasts	NCT02032225
36	ClinicalTrials.gov	Derivation of New Human Embryonic Stem Cell Lines: Identification of Instructive Factors for Germ Cells Development	NCT01165918
37	ClinicalTrials.gov	A Phase I/IIa, Open-Label, Single-Center, Prospective Study to Determine the Safety and Tolerability of Sub-retinal Transplantation of Human Embryonic Stem Cell Derived Retinal Pigmented Epithelial(MA09-hRPE) Cells in Patients With Advanced Dry Age-related Macular Degeneration(AMD)	NCT01674829
38	ClinicalTrials.gov	Stem Cell Therapy for Outer Retinal Degenerations	NCT02903576
39	ClinicalTrials.gov	CIQTP Prolongation : Role and Mechanism in Sudden Cardiac Death (IQARE-SCD)	NCT03387072
40	ClinicalTrials.gov	Establishment of Genetic Basis for Neurological Disease by Genetic Screening	NCT03322306
41	ClinicalTrials.gov	Malignant Hyperthermia Registry and Genetic Testing	NCT02964481
42	ClinicalTrials.gov	Safety and Efficacy Study of Human ESC-derived Neural Precursor Cells in the Treatment of Parkinson's Disease	NCT03119636
43	ClinicalTrials.gov	BAP-EB as a Predictive Tool for Endometrial Receptivity and Pregnancy Outcome of IVF Treatment	NCT02713854
44	ClinicalTrials.gov	UTHealth Turner Syndrome Research Registry	NCT03185702
45	ClinicalTrials.gov	The Crnic Institute Human Trisome Project Biobank (HTP)	NCT02864108
46	ClinicalTrials.gov	Safety and Efficacy Study of OpRegen for Treatment of Advanced Dry-Form Age-Related Macular Degeneration	NCT02286089
47	ClinicalTrials.gov	Safety and Tolerability of MA09-hRPE Cells in Patients With Stargardt's Macular Dystrophy(SMD)	NCT01625559
48	ClinicalTrials.gov	Ability of a Molecule (Prima) to Restore Physiological Differentiation in Epithelium Expressing Gene p63 (PRIMAculture)	NCT02896387
49	ClinicalTrials.gov	Mesenchymal Stem Cells (MSCs) - Like Cell Transplantation in Women With Primary Ovarian Insufficiency (MSCLCTWPOI)	NCT03877471
50	ClinicalTrials.gov	Latent Structure of Multi-level Assessments and Predictors of	NCT02450240

		Outcomes in Psychiatric Disorders	
51	ClinicalTrials.gov	The Role of TBX3 in Human ES Cell Differentiation	NCT00581152
52	ClinicalTrials.gov	Early MRI Detection of Myocardial Deterioration as a Preventive, Disease Staging, and Prognostic Biomarker in Insulin Resistance	NCT03509441
53	ClinicalTrials.gov	Heart and Muscle Metabolism in Barth Syndrome	NCT01625663
54	ClinicalTrials.gov	Alpha-1 Antitrypsin Deficiency Adult Liver Study	NCT02014415
55	ClinicalTrials.gov	FT500 as Monotherapy and in Combination With Immune Checkpoint Inhibitors in Subjects With Advanced Solid Tumors	NCT03841110
56	ClinicalTrials.gov	Differences in Peritoneal Stem Cells in Women With and Without Adhesions After Gynaecological Surgery	NCT02847676
57	ClinicalTrials.gov	Genetic and Environmental Determinants That Control Metabolism in Pulmonary Hypertension	NCT02594917
58	ClinicalTrials.gov	Whole Transcriptome Profiling and Metabolic Phenotyping in Children With ROHHAD Syndrome	NCT02602769
59	ClinicalTrials.gov	Rare CFTR Mutation Cell Collection Protocol (RARE) (RARE)	NCT03161808
60	ClinicalTrials.gov	In Vitro Model of the Cystic Fibrosis Bronchial Epithelium Via iPS Technology (PaCyFIC)	NCT03754088
61	ClinicalTrials.gov	Developing Protocols for Modelling of Genetic Diseases Using Induced Pluripotent Stem Cells	NCT03612310
62	ClinicalTrials.gov	Generation of Cancer Antigen-Specific T-cells From Human Induced Pluripotent Stem Cells (iPSC) for Research and Potential FutureTherapy	NCT03407040
63	ClinicalTrials.gov	Development of the Tool " iPSC " for the Functional Study of Mutations Responsible for Mental Retardation (Rementips)	NCT02980302
64	ClinicalTrials.gov	Feasibility of Generating Pluripotent Stem Cells From Patients With Familial Retinoblastoma	NCT02193724
65	ClinicalTrials.gov	Pharmacogenomic Evaluation of Antihypertensive Responses in Induced Pluripotent Stem (iPS) Cells Study (PEAR-iPSC)	NCT01943383
66	ClinicalTrials.gov	Cell-Based Approaches For Modeling and Treating Ataxia-Telangiectasia	NCT02246491
67	ClinicalTrials.gov	Induction of Pluripotent Stem Cells	NCT02084407

		From Human Fibroblasts of DM1 Patients	
68	ClinicalTrials.gov	Derivation of Human Induced Pluripotent Stem (iPS) Cells to Heritable Cardiac Arrhythmias	NCT02413450
69	ClinicalTrials.gov	Evaluating Cardiovascular Phenotypes Using Induced Pluripotent Stem Cells (iPSC)	NCT01517425
70	ClinicalTrials.gov	Stem Cell Study of Genetics and Drug Addiction	NCT01534624
71	ClinicalTrials.gov	Molecular Mechanism Identification in Inherited Arrhythmias and Valvulopathies From Induced Pluripotent Stem Cells (Diag-iPS)	NCT01734356
72	ClinicalTrials.gov	Transplantation of Human Embryonic Stem Cell-derived Progenitors in Severe Heart Failure (ESCORT)	NCT02057900
73	ClinicalTrials.gov	Generation of Powerful Biological Tools for Understanding the Pathophysiology of Chronic Granulomatous Disease. (FIBRO CGD)	NCT02926963
74	ClinicalTrials.gov	Stem Cell Models of Best Disease and Other Retinal Degenerative Diseases.	NCT02162953
75	ClinicalTrials.gov	Individualized Early Risk Assessment for Heart Diseases (IndivuHeart)	NCT02417311
76	ClinicalTrials.gov	Skin and Blood Research Samples From Healthy Volunteers and Patients With Hematologic Diseases	NCT00840567
77	ClinicalTrials.gov	Answer ALS: Individualized Initiative for ALS Discovery (AnswerALS)	NCT02574390
78	ClinicalTrials.gov	A Safety Surveillance Study in Subjects With Macular Degenerative Disease Treated With Human Embryonic Stem Cell-derived Retinal Pigment Epithelial Cell Therapy	NCT03167203
79	ClinicalTrials.gov	CAUSE Trial: Patient Specific-Cellular Characterization of Fibromuscular Dysplasia and High-Risk Atherosclerotic Endothelium	NCT01808729
80	ClinicalTrials.gov	Patient Specific Induced Pluripotency Stem Cells (PSiPS)	NCT00953693
81	ClinicalTrials.gov	Translational Approaches to Septic Cardiomyopathy (TASC01)	NCT03252613
82	ClinicalTrials.gov	Generation of Induced Pluripotent Stem (iPS) Cell Lines From Skin Fibroblast Cells of Participants With Age-Related Macular Degeneration	NCT03372746
83	ClinicalTrials.gov	A Phase I Study of iPS Cell Generation From Patients With COPD	NCT01860898

84	ClinicalTrials.gov	Creation of a Bank of Fibroblast From Patients With Amyotrophic Lateral Sclerosis: Pilot Study (ALSCELL)	NCT01639391
85	ClinicalTrials.gov	Establishing Fibroblast-Derived Cell Lines From Skin Biopsies of Patients With Immunodeficiency or Immunodysregulation Disorders	NCT00895271
86	ClinicalTrials.gov	Investigating Hereditary Cardiac Disease by Reprogramming Skin Cells to Heart Muscle (CLUE)	NCT01865981
87	ClinicalTrials.gov	Study of Subretinal Implantation of Human Embryonic Stem Cell-Derived RPE Cells in Advanced Dry AMD	NCT02590692
88	ClinicalTrials.gov	Study of Neurodegenerative Diseases Induced Stem Cells in Patients and Healthy Family Controls. (NeuronsiPS)	NCT03682458
89	ClinicalTrials.gov	Pathology of Helicases and Premature Aging: Study by Derivation of hiPS (HeliPS)	NCT03898817
90	ClinicalTrials.gov	Interventional Study of Implantation of hESC-derived RPE in Patients With RP Due to Monogenic Mutation	NCT03963154
91	ClinicalTrials.gov	A Follow up Study to Determine the Safety and Tolerability of Sub-retinal Transplantation of Human Embryonic Stem Cell Derived Retinal Pigmented Epithelial (hESC-RPE) Cells in Patients With Stargardt's Macular Dystrophy (SMD)	NCT02941991
92	ClinicalTrials.gov	Metformin as a Chemoprevention Agent in Non-small Cell Lung Cancer	NCT01717482
93	ClinicalTrials.gov	The Safety and Tolerability of Sub-retinal Transplantation of SCNT-hES-RPE Cells in Patients With Advanced Dry AMD	NCT03305029
94	ClinicalTrials.gov	Safety and Tolerability of Sub-retinal Transplantation of Human Embryonic Stem Cell Derived Retinal Pigmented Epithelial (hESC-RPE) Cells in Patients With Stargardt's Macular Dystrophy (SMD)	NCT01469832
95	ClinicalTrials.gov	Modeling the Neurological Basis and Characterizing the Neurological Phenotype of Obesity Using Human Neural Stem Cells	NCT03263390
96	ClinicalTrials.gov	Target Validation and Discovery in Idiopathic Bronchiectasis	NCT03750734
97	ClinicalTrials.gov	Safety Observation on hESC Derived MSC Like Cell for the Meniscus Injury	NCT03839238
98	ClinicalTrials.gov	A Study of CYP-001 for the Treatment of Steroid-Resistant Acute Graft	NCT02923375

		Versus Host Disease	
99	ClinicalTrials.gov	Sub-retinal Transplantation of hESC Derived RPE(MA09-hRPE)Cells in Patients With Stargardt's Macular Dystrophy	NCT01345006
100	ClinicalTrials.gov	Safety and Tolerability of Sub-retinal Transplantation of hESC Derived RPE (MA09-hRPE) Cells in Patients With Advanced Dry Age Related Macular Degeneration (Dry AMD)	NCT01344993
101	ClinicalTrials.gov	Long Term Follow Up of Sub-retinal Transplantation of hESC Derived RPE Cells in Stargardt Macular Dystrophy Patients	NCT02445612
102	ClinicalTrials.gov	Long Term Follow Up of Sub-retinal Transplantation of hESC Derived RPE Cells in Patients With AMD	NCT02463344
103	ClinicalTrials.gov	A Study Of Implantation Of Retinal Pigment Epithelium In Subjects With Acute Wet Age Related Macular Degeneration	NCT01691261
104	ClinicalTrials.gov	Retinal Pigment Epithelium Safety Study For Patients In B4711001	NCT03102138
105	ClinicalTrials.gov	Biomarkers for Pain in Spinal Cord Injury (SCI) Patients (SCI Pain)	NCT00913471
106	ClinicalTrials.gov	Assessment of Paclitaxel-Induced Neuropathy	NCT01953159
107	ClinicalTrials.gov	Understanding Clinical Phenotype and Collecting Biomarker Samples in C9ORF72 ALS	NCT02686268
108	ClinicalTrials.gov	Clinical Study of Subretinal Transplantation of Human Embryo Stem Cell Derived Retinal Pigment Epitheliums in Treatment of Macular Degeneration Diseases	NCT02749734
109	ClinicalTrials.gov	Studies of Autistic Patients: Gene Networks and Clinical Subtypes	NCT01092208
110	ICTRP	MSC-like cell therapy for primary ovarian insufficiency	ChiCTR1900021921
111	ICTRP	Kyoto Trial to Evaluate the Safety and Efficacy of iPSC-derived dopaminergic progenitors in the treatment of Parkinson's Disease	JPRN-UMIN000033564
112	ICTRP	Kyoto Trial to Evaluate the Safety and Efficacy of Tacrolimus in the iPSC-based Therapy for Parkinson's Disease	JPRN-UMIN000033565
113	ICTRP	Clinical trial of human (allogeneic) induced pluripotent stem cell-derived cardiomyocyte sheet for severe	JPRN-UMIN000032989

		cardiomyopathy	
114	ICTRP	Understanding of vitiligo (skin disease).	CTRI/2018/04/013393
115	ICTRP	Individualized induced pluripotent stem cells from the fibroblasts of patients' lesions with different skin diseases	ChiCTR-BOC-17011649
116	ICTRP	A Study of transplantation of allogenic induced pluripotent stem cell (iPSC) derived retinal pigment epithelium (RPE) cell suspension in subjects with neovascular age related macular degeneration	JPRN-UMIN000026003
117	ICTRP	Generation of induced pluripotent stem cells (iPSCs) derived from intractable retinal disease patients and clarification of the diseases and novel therapies development by using retinal tissues differentiated and induced from iPSC.	JPRN-UMIN000021556
118	ICTRP	Investigation of the underlying pathomechanisms found in defects of the neurotransmitter, Pterine -, phenyl alanine, and 5-Methyltetrahydrofolate metabolism in induced pluripotent stem cells (iPSC) and derivatives	DRKS00010150
119	ICTRP	Basic research of immunotherapy using dendritic cells derived from induced pluripotent stem cells in healthy donors and cancer patients	JPRN-UMIN000021105
120	ICTRP	Establishment and analyses of induced pluripotent stem (iPS) cells / human-induced neuronal (hiN) cells / induced microglia-like (iMG) cells from tissue, to elucidate the pathogenesis and pathophysiology of mental and developmental disorders.	JPRN-UMIN000020468
121	ICTRP	Generation of induced pluripotent stem cells from somatic cells of healthy individuals and patients with inherited heart diseases	DRKS00009433
122	ICTRP	Clinical study of subretinal transplantation of clinical human embryonic stem cells derived retinal pigment epitheliums in treatment of dry age-related macular degeneration diseases	ChiCTR-OCB-15007054
123	ICTRP	Analysis of diseases in otorhinolaryngology using iPS cells	JPRN-UMIN000018051
124	ICTRP	Next Generation Sequencing and Induced Pluripotent Stem Cell Applications in Genetic and Inheritable	ACTRN12615000140550

		Forms of Renal Disease	
125	ICTRP	Induced pluripotent stem cell (iPSC)-derived immortalized megakaryocyte cell lines (imMKCLs) from voluntary blood component donors with specific HLA haplotypes towards platelet products in transfusion therapy.	JPRN-UMIN000015345
126	ICTRP	The clinical trial of human embryonic stem cell derived epithelial cells transplantation in the treatment of severe ocular surface diseases	ChiCTR-OCB-15005968
127	ICTRP	A Study of transplantation of autologous induced pluripotent stem cell (iPSC) derived retinal pigment epithelium (RPE) cell sheet in subjects with exudative age related macular degeneration	JPRN-UMIN000011929
128	ICTRP	Development of human cellular models for motor neuron diseases using disease-specific induced pluripotent stem (iPS) cells	JPRN-UMIN000011542
129	ICTRP	Development of induced pluripotent stem (iPS) cells from patients with muscular disorders for the study of the pathogenesis of the diseases and development of therapies	JPRN-UMIN000008958
130	ICTRP	Plasticity in prefrontal cortical regions in humans: genetic variation, cellular mechanisms and modulation by means of neurofeedback	DRKS00004252
131	ACTRN	The Western Australia Retinal Degeneration Study: An natural history observational cohort study of retinal degenerations and in vitro retinal disease modelling using patient derived stem cells	ACTRN12618000738224

Supplementary Table 1. List of all studies involving pluripotent stem cells

ID number	Title	Cell type	Origin	Target disease	Organ	Country or Area	Study design	Study phase	No. patients enrolled	Study groups	Intervention	Status
NCT03222453	Thalassemia Treatment Based on the Stem Cell Technology	hiPSC	autologous	Beta-Thalassemia	Hematologic disorder	China	Open Label	N.A.	2	Intervention/ No Intervention	Injection of hematopoietic stem cells	Results submitted to ClinicalTrials.gov
NCT01674829	A Phase I/IIa, Open-Label, Single-Center, Prospective Study to Determine the Safety and Tolerability of Sub-retinal Transplantation of Human Embryonic Stem Cell Derived Retinal Pigmented Epithelial(MA09-hRPE) Cells in Patients With Advanced Dry Age-related Macular Degeneration	hESC-derived RPE; MA09-hRPE	allogeneic	Dry Age Related Macular Degeneration	Sense organ diseases	South Korea	Open Label	Phase I/IIa	12	4 groups, different number of cells	Sub-retinal Transplantation	Active, not recruiting
NCT02903576	Stem Cell Therapy for Outer Retinal Degenerations	hESC-derived RPE	allogeneic	Age Related Macular Degeneration, Stargardt's Disease or Exudative Age-related Macular Degeneration	Sense organ diseases	Brazil	Open Label	Phase I/II	18	2 active comparators	Injection of hESC-RPE in suspension versus injection of hESC-RPE seeded in a substrate	Not yet reported, recruitment status unknown
NCT01625559	Safety and Tolerability of MA09-hRPE Cells in Patients With Stargardt's Macular Dystrophy(SMD)	hESC-derived RPE cells (MA09-hRPE)	allogeneic	Stargardt's Macular Dystrophy	Sense organ diseases	South Korea	Open Label	Phase I	3	Single arm	Sub-retinal transplantation	Not yet reported, recruitment status unknown
NCT03763136	Treating Heart Failure With hPSC-CMs	hiPSCs-derived cardiomy	allogeneic	Heart Failure	Cardiovascular diseases	China	Open Label	NA	5	Single arm	Injection into the myocardium	Recruiting

		ocytes										
NCT03046407	Treatment of Dry Age Related Macular Degeneration Disease With Retinal Pigment Epithelium Derived From Human Embryonic Stem Cells	hESC-derived RPE	allogeneic	Dry Age-related Macular Degeneration	Sense organ diseases	China	Open Label	Phase I/II	10	Single arm	Subretinal transplantation	Recruiting
NCT03944239	Safety and Efficacy of Subretinal Transplantation of Clinical Human Embryonic Stem Cell Derived Retinal Pigment Epitheliums in Treatment of Retinitis Pigmentosa	hESC-derived RPE	allogeneic	Retinitis pigmentosa	Sense organ diseases	China	Open Label	Phase I	10	Single arm	Subretinal transplantation	Recruiting
NCT03482050	Study to Evaluate Transplantation of Astrocytes Derived From Human Embryonic Stem Cells, in Patients With Amyotrophic Lateral Sclerosis	hESC-derived astrocytes	allogeneic	Amyotrophic Lateral Sclerosis	Neurological disorders	Israel	Open Label	Phase I/IIa	21	Dose-escalation, 4 groups	intrathecal (spinal) injection	Recruiting
NCT02755428	Subretinal Transplantation of Retinal Pigment Epitheliums in Treatment of Age-related Macular Degeneration Diseases	hESC-derived RPE	allogeneic	Dry Age-related Macular Degeneration	Sense organ diseases	China	Open Label	Phase I/II	10	Single arm	Subretinal transplantation	Recruiting
NCT03119636	Safety and Efficacy Study of Human ESC-derived Neural Precursor Cells in the Treatment of Parkinson's Disease	hESC-derived neural precursor cells	allogeneic	Parkinson's disease	Neurological disorders	China	Open Label	Phase I/II	50	3 groups: Levodopa 1) +NPC 2) + HLA-matched NPC, 3) + HLA-non-matched	Stereotaxic intra-striatal injection	Recruiting

										NPC		
NCT02286089	Safety and Efficacy Study of OpRegen for Treatment of Advanced Dry-Form Age-Related Macular Degeneration	hESC-derived RPE	allogeneic	Age-related Macular Degeneration	Sense organ diseases	USA+Israel	Open Label	Phase I/IIa	24	Dose-escalation, 4 groups	Subretinal transplantation	Recruiting
NCT03877471	Mesenchymal Stem Cells - Like Cell Transplantation in Women With Primary Ovarian Insufficiency	hESC-derived MSC-like cells	allogeneic	Primary Ovarian Insufficiency	Reproductive diseases	China	Open Label	Phase I	28	3 groups: low, medium and high dosage of cells	Injection into ovaries bilaterally	Recruiting
NCT03841110	FT500 as Monotherapy and in Combination With Immune Checkpoint Inhibitors in Subjects With Advanced Solid Tumors	iPSC-derived NK cell cancer immunotherapy	allogeneic	advanced solid tumors	Neoplasms	USA	Open Label	Phase I	76	1) FT500 Monotherapy, 2) FT500 in Combination with Immune Checkpoint Inhibitor nivolumab, pembrolizumab or atezolizumab		Recruiting
JPRN-UMIN000032989	Clinical trial of human (allogeneic) induced pluripotent stem cell-derived cardiomyocyte sheet for severe cardiomyopathy	iPS cell-derived cardiomyocytes	allogeneic	Ischemic cardiomyopathy	Cardiovascular diseases	Japan	Open Label	Phase I, II	3	Single arm	iPS cell-derived cardiomyocyte sheet transplantation	Recruitment complete
NCT03963154	Interventional Study of Implantation of hESC-derived RPE in Patients With RP Due to Monogenic Mutation	hESC-derived RPE	allogeneic	Retinitis Pigmentosa Due to Monogenic Mutation	Sense organ diseases	France	Open Label	Phase I/II	12	2 sequential cohorts: 1) very advanced and 2) less advanced loss of visual acuity	single central subretinal implantation of a hESC-derived RPE monolayer in one eye	Recruiting
NCT03305029	The Safety and Tolerability of Sub-retinal Transplantation of SCNT-hES-RPE	SCNT hESC-derived RPE	allogeneic	Dry Age-Related Macular Degeneration	Sense organ diseases	South Korea	Open-Label	Phase I	3	Single arm	Subretinal transplantation	Recruitment status unknown

	Cells in Patients with Advanced Dry AMD											
NCT02057900	Transplantation of Human Embryonic Stem Cell-derived Progenitors in Severe Heart Failure (ESCORT)	hESC-derived CD15+ Isl-1+ progenitors	allogeneic	Ischemic Heart Disease	Cardiovascular diseases	France	Open-Label	Phase I	10	Single arm	fibrin gel embedding hESC-derived CD15+ Isl-1+ progenitors	Completed, results published [1]
NCT01469832	Safety and Tolerability of Sub-retinal Transplantation of Human Embryonic Stem Cell Derived Retinal Pigmented Epithelial (hESC-RPE) Cells in Patients with Stargardt's Macular Dystrophy (SMD)	hESC-derived RPE cells (MA09-hRPE)	allogeneic	Stargardt's Macular Dystrophy	Sense organ diseases	UK	Open-Label, Multi-Center	Phase I/II	12	Dose-escalation; 5 cohorts - 4 low vision cohorts and 1 better vision cohort	Subretinal injection	Completed, results published [2]
NCT01345006	Sub-retinal Transplantation of hESC Derived RPE(MA09-hRPE)Cells in Patients With Stargardt's Macular Dystrophy	hESC-derived RPE cells (MA09-hRPE)	allogeneic	Stargardt's Macular Dystrophy	Sense organ diseases	USA	Open-Label, Multi-Center	Phase I/II	13	Dose-escalation; 5 cohorts - 4 low vision cohorts and 1 better vision cohort	Subretinal injection	Completed, results published [3,4]
NCT01344993	Safety and Tolerability of Sub-retinal Transplantation of hESC Derived RPE (MA09-hRPE) Cells in Patients With Advanced Dry Age Related Macular Degeneration	hESC-derived RPE cells (MA09-hRPE)	allogeneic	Dry Age-Related Macular Degeneration	Sense organ diseases	USA	Open-Label, Multi-Center	Phase I/II	13	Dose-escalation; 5 cohorts - 4 low vision cohorts and 1 better vision cohort	Subretinal injection	Completed, results published [3,4]
JPRN-UMIN000011929	A Study of transplantation of autologous induced pluripotent stem cell (iPSC) derived retinal pigment epithelium	hiPSC-derived RPE	autologous	Exudative age-related macular degeneration	Sense organ diseases	Japan	Open Label		6	Single arm	Transplantation of iPSC-derived RPE cell sheet	Complete, results published[5]

	(RPE) cell sheet in subjects with exudative age related macular degeneration											
NCT02590692	Study of Subretinal Implantation of Human Embryonic Stem Cell-Derived RPE Cells in Advanced Dry AMD	hESC-derived RPE	allogeneic	Dry Age-Related Macular Degeneration	Sense organ diseases	USA	Open Label	Phase I/IIa	16	Treated eye and non-treated eye	Subretinal implantation	Active, not recruiting
NCT03839238	Safety Observation on hESC Derived MSC Like Cell for the Meniscus Injury	hESC-derived MSC like cell	allogeneic	Meniscus injury	Musculoskeletal disorders	China	Open Label	Phase I	18	Three cohorts with different dosages	hESC-derived MSC like cells	Active, not recruiting
NCT02923375	A Study of CYP-001 for the Treatment of Steroid-Resistant Acute Graft Versus Host Disease	iPSC-derived MSCs	allogeneic	Graft vs Host Disease	Immune System	Australia + UK	Open Label	Phase I	16	Dose escalation, 2 groups	IV infusion on two occasions	Active, not recruiting
NCT01691261	A Study Of Implantation Of Retinal Pigment Epithelium In Subjects With Acute Wet Age Related Macular Degeneration	hESC-derived RPE	allogeneic	Age-Related Macular Degeneration	Sense organ diseases	UK	Open Label	Phase I	2	Single arm	Implantation of RPE monolayer immobilized on a polyester membrane.	Active, not recruiting
NCT02749734	Clinical Study of Subretinal Transplantation of Human Embryo Stem Cell Derived Retinal Pigment Epitheliums in Treatment of Macular Degeneration Diseases	hESC-derived RPE	allogeneic	Macular Degeneration Stargardt's Macular Dystrophy	Sense organ diseases	China	Open label	Phase I/II	15	Single arm	Subretinal transplantation	Recruitment status unknown
JPRN-UMIN000033564	Kyoto Trial to Evaluate the Safety and Efficacy of iPSC-derived dopaminergic progenitors in the treatment of	hiPSC-derived dopaminergic progenitors		Parkinson's Disease	Sense organ diseases	Japan	Open Label	Phase I/II	7	Single arm	Transplantation into the corpus striatum	Recruitment suspended

	Parkinson's Disease											
JPRN-UMIN000026003	A Study of transplantation of allogenic induced pluripotent stem cell (iPSC) derived retinal pigment epithelium (RPE) cell suspension in subjects with neovascular age related macular degeneration	iPSC-derived RPE	allogeneic	Neovascular age related macular degeneration	Sense organ diseases	Japan	Open Label		5	Single arm	Subretinal transplantation	Recruitment complete; follow-up continuing
ChiCTR-OCB-15007054	Clinical study of subretinal transplantation of clinical human embryonic stem cells derived retinal pigment epitheliums in treatment of dry age-related macular degeneration diseases	hESC-derived RPE	allogeneic	Dry age-related macular degeneration	Sense organ diseases	China	Open Label		10	Single arm	Subretinal transplantation	Recruiting
ChiCTR-OCB-15005968	The clinical trial of human embryonic stem cell derived epithelial cells transplantation in the treatment of severe ocular surface diseases	hESC-derived epithelial cells	allogeneic	Severe Ocular Surface Disease	Sense organ diseases	China	Open Label		20	Historical control	Corneal-epithelium-like-cell transplantation	Recruiting

Supplementary Table 2. Characteristics of all interventional studies. Abbreviations: hESC = human embryonic stem cells, hiPSC = human induced pluripotent stem cell, RPE = retinal pigment epithelium, hRPE = human retinal pigment epithelium, MSC = mesenchymal stem cell, UK = United Kingdom

Supplementary References

1. Menasché, P., *et al.* Transplantation of Human Embryonic Stem Cell-Derived Cardiovascular Progenitors for Severe Ischemic Left Ventricular Dysfunction. *Journal of the American College of Cardiology* **71**, 429-438 (2018).
2. Mehat, M.S., *et al.* Transplantation of Human Embryonic Stem Cell-Derived Retinal Pigment Epithelial Cells in Macular Degeneration. *Ophthalmology* **125**, 1765-1775 (2018).
3. Schwartz, S.D., *et al.* Embryonic stem cell trials for macular degeneration: a preliminary report. *Lancet (London, England)* **379**, 713-720 (2012).
4. Schwartz, S.D., *et al.* Human embryonic stem cell-derived retinal pigment epithelium in patients with age-related macular degeneration and Stargardt's macular dystrophy: follow-up of two open-label phase 1/2 studies. *Lancet (London, England)* **385**, 509-516 (2015).
5. Mandai, M., *et al.* Autologous Induced Stem-Cell-Derived Retinal Cells for Macular Degeneration. *The New England journal of medicine* **376**, 1038-1046 (2017).
